# Distribution of Herbivorous Fish Is Frozen by Low Temperature

**DOI:** 10.1038/srep39600

**Published:** 2016-12-22

**Authors:** Ivana Vejříková, Lukáš Vejřík, Jari Syväranta, Mikko Kiljunen, Martin Čech, Petr Blabolil, Mojmír Vašek, Zuzana Sajdlová, Son Hoang The Chung, Marek Šmejkal, Jaroslava Frouzová, Jiří Peterka

**Affiliations:** 1 Biology Centre of the Czech Academy of Sciences, Institute of Hydrobiology, Na Sádkách 7, 37005 České Budějovice, Czech Republic; 2 Faculty of Science, University of South Bohemia in České Budějovice, Branišovská 31, 37005 České Budějovice, Czech Republic; 3 Department of Bioscience, Aarhus University, Vejlsøvej 25, 8600 Silkeborg, Denmark; 4 Department of Environmental and Biological Sciences, University of Eastern Finland, P.O. Box 111, FI-80101 Joensuu, Finland; 5 Department of Biological and Environmental Science, University of Jyväskylä, P.O. Box 35, FI-40014 University of Jyväskylä, Finland

## Abstract

The number of herbivores in populations of ectothermic vertebrates decreases with increasing latitude. At higher latitudes, fish consuming plant matter are exclusively omnivorous. We assess whether omnivorous fish readily shift to herbivory or whether animal prey is typically preferred. We address temperature as the key factor causing their absence at higher latitudes and discuss the potential poleward dispersion caused by climate changes. A controlled experiment illustrates that rudd (*Scardinius erythrophthalmus*) readily utilize plant matter at water temperatures above 20 °C and avoid its consumption below 20 °C. Field data support these results, showing that plant matter dominates rudd diets during the summer and is absent during the spring. Utilizing cellulose requires the enzyme cellulase, which is produced by microorganisms growing at temperatures of 15–42 °C. Water temperatures at higher latitudes do not reach 15 °C year-round; at our latitude of 50°N~150 days/year. Hence, the species richness of omnivorous fish decreases dramatically above 55° latitude. Our results provide support for the hypothesis that strict herbivorous specialists have developed only in the tropics. Temperatures below 15 °C, even for a short time period, inactivate cellulase and cause diet limitations for omnivorous fish. However, we may expect increases in herbivory at higher latitudes caused by climate change.

A specialization of herbivory in aquatic ecosystems was previously considered a sporadic phenomenon with irrelevant impacts on the water communities^1^,^2^. However, recent studies indicate that herbivory in aquatic ecosystems has been overlooked by scientists in comparison to terrestrial ecosystems^3^,^4^,^5^,^6^, and the impact of herbivory by both vertebrates and invertebrates on aquatic ecosystems is substantial^7^,^8^,^9^. Fishes have the following two methods of digesting barely degradable high molecular weight polysaccharide material: i) predominantly chemical processing by acid hydrolysis in the stomach (e.g., Acanthuridae, Kyphosidae, Pomacentridae) or ii) mechanical processing by breakdown of plant matter using a muscular stomach (e.g., Acanthuridae, Sparidae) or pharyngeal teeth (e.g., Cichlidae, Cyprinidae, Scaridae)^10^,^11^,^12^.

A specialization in herbivory is often developed by fish and other ectothermic aquatic and terrestrial vertebrates at lower latitudes^13^,^14^. In contrast, ectothermic vertebrates (mainly fish) at higher latitudes that use plant matter are omnivores, consuming both plant and animal sources in their diets. At these latitudes, either a herbivorous specialization has not developed or evidence of this type of specialization is lacking in the fossil record^14^,^15^,^16^. The quantity of plant matter in the diet of omnivores and the number of ectothermic species capable of consuming plant matter rapidly decrease poleward^16^,^17^,^18^,^19^, which can potentially be explained by a number of theories as follows: (i) short-term evolution and the inability of fish to migrate along latitudinal gradients^20^, (ii) availability of a readily palatable plant diet at lower latitudes^21^, (iii) absence of plant diet at higher latitudes during the winter^22^, and (iv) constraints in the digestion of plant matter at low temperatures^14^,^18^,^23^. The last theory is supported by observations that marine herbivorous fish have spread to higher latitudes due to global warming^24^,^25^.

Of the European native fish species found in lentic water, herbivory is most developed in rudd (*Scardinius erythrophthalmus*). The proportion of plant-based diet consumed (mainly macrophytes) is markedly higher for rudd than for other species, e.g., roach (*Rutilus rutilus*), even when a nutrient-rich diet is available^26^. Recent studies examining macrophyte characteristics and the causes of selective herbivory in rudd assert that the greatest factors are C:N ratio and phenolic concentration, both of which are negatively correlated with herbivory rates. Lesser importance is assigned to dry matter content and the concentration of total soluble proteins^15^,^27^,^28^. The shared evolution of rudd and macrophytes also seems to be a relevant factor^28^.

Although rudd are shown to prefer an animal diet in experimental conditions (aquarium, mesocosm)^7^,^15^, natural observations indicate a preference for a plant-dominated diet in the wild^29^,^30^. The percentage of plant matter in rudd diet usually increases with size and age^31^. However, the key environmental factor driving these preferences is still unknown. According to previous findings, temperature seems to play an important role, as it is positively correlated with the intake of plant matter by omnivorous fish^14^,^26^,^30^,^32^. This trend has also been observed in other fish species^33^. It is often a consequence of changes in other environmental factors, such as plant availability^22^ and the increased cellulolytic activity mediated by symbiotic microorganisms^10^,^30^.

This study focuses on the food preferences of omnivorous fish using rudd as a model organism in two oligotrophic lakes exhibiting differences in both macrophyte occurrence and animal food availability. Our principal conclusions were drawn from mesocosm experiments testing rudd preferences for animal prey versus plant matter in the presence of different concentrations of food and under a range of temperature conditions (16, 20, 24 °C). The primary aim of the study was to determine if omnivorous fish readily shift to herbivory, or whether animal prey is always preferred. These results will enhance our understanding of the key factors causing the absence of ectothermic herbivores at higher latitudes. Additionally, recent climate change models predict rapid warming, particularly at higher latitudes. The results of this study can provide important insights into how many herbivorous fish species may disperse to new areas as water temperatures increase and fish species gain advantages in trophic competition by utilizing resources that are not available to other aquatic animals, including native species.

## Results

### An assessment of potential diet for rudd in studied lakes

In both lakes, macrophytes and macroalgae (*Chara* and *Vaucheria*, henceforth referred to as macrophyte coverage for simplicity) occurred down to a depth of 12 m, but the abundance in the two locations differed noticeably. In Milada Lake in September, dense coverage by submerged macrophytes reached 91% at the primary rudd habitat depth of 0–3 m, with a mean wet mass of 2,720 g m^−2^ (Table 1). In May, the coverage was lower (60.1%), but macrophytes were still present with a mean mass of 1,275 g m^−2^. The species occurring at this depth were as follows: *Potamogeton pectinatus* (September: 40%; May: 30%), *Myriophyllum spicatum* (20%; 12%), *Chara* sp. (19%; 11%), *Vaucheria* (11.5%; 7%), *Potamogeton trichoides* (0.2%; 0%), *Spharganium emersum* (0.15%; 0%)*, Myriophyllum verticillatum* (0.05%; 0%), *Potamogeton crispus* (0.05%; 0.1%) and *Elodea canadensis* (0.04%; 0%). In Most Lake, there was only a sparse macrophyte coverage of 1.6% and 0.1% at a depth of 0–3 m, and a mean mass of only 51 g m^−2^ and 11 g m^−2^ in September and May, respectively. The species occurring at this depth were *Myriophylum spicatum* (September: 0.8%; May: 0.03%), *Potamogeton pectinatus* (0.3%; 0.04%), *Chara* sp. (0.2%; 0.03%), *Spharganium emersum* (0.2%; 0%) and *Potamogeton crispus* (0.1%; 0%). The shorelines of both lakes comprise stones covered by periphyton layers of similar densities, but an accurate biomass measurement was not taken.

Invertebrates living in the fine sediment, on the surface of the stones and macrophytes had a mean biomass 5.4 g m^−2^ and 4.2 g m^−2^ in Milada and Most Lakes, respectively. In both lakes, the following genera occurred in order of descending biomass: waterlouse (*Asellus aquaticus*), dragon fly larvae (Odonata), chironomid larvae (*Chironomus* spp.) and caddisfly larvae (Trichoptera). Additionally, zebra mussels (*Dreissena polymorpha*) occurred at high densities in both studied lakes and were the only potential rudd food source that was more abundant in Most Lake than in Milada Lake (Table 1). However, this food source was not found in rudd stomachs in either of the studied lakes (Table 2).

The mean density of zooplankton at 0–20 m depth was 36.3 ind. L^−1^ and 27.4 ind. L^−1^ in Milada and Most Lakes, respectively. Copepods (Copepoda) and large *Daphnia* were more abundant in Milada Lake, while small *Daphnia* were more abundant in Most Lake (Table 1).

### Diet results revealed by Gut Content Analysis (GCA) and Stable Isotope Analysis (SIA)

GCA revealed that plant matter dominated the diet of rudd in September 2013 and 2014 (surface water temperature: 19.1–21 °C) in both lakes. For rudd older than one year in Milada Lake, 92.5% of the food consumed was plant matter in the form of macrophytes, and the rest was animal prey (Table 2). In Most Lake, plant matter also dominated but consisted of periphyton (68%) and detritus (25%). Animal prey accounted for 7% of the gut contents. In September in Milada Lake, juvenile rudd were strict herbivores consuming macrophytes, whereas in Most Lake they were strict zooplanktivores. In contrast to the warmer month of September, in May 2015 (surface water temperature: 13.1–14.2 °C), only animal prey was found in rudd digestive tracts. In Milada Lake, diet consisted solely of the aerial stage of aquatic insects. In Most Lake, it consisted of the aerial stage of aquatic insects, benthic invertebrates and zooplankton (Table 2).

**Figure 1:**
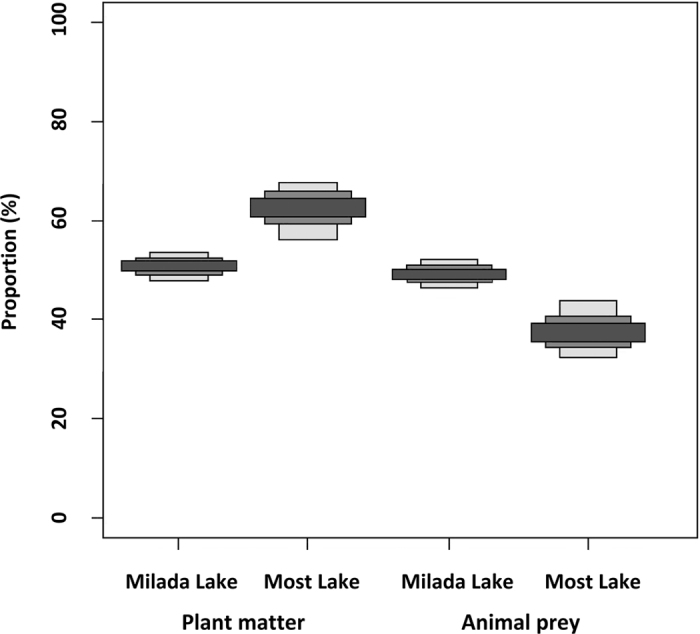
Probability proportion of plant matter and animal prey in assimilated diet of rudd in Milada and Most Lakes according to stable isotope analysis. Plant matter is presented by the categories macrophytes (Milada Lake), periphyton and detritus (both Most Lake). Animal prey is presented by the categories zooplankton, benthos, and zebra mussel (*Dreissena polymorpha*). The credibility intervals are 95, 75 and 25%. (see Supplementary Fig. S3 for SIA Biplots).

The results of SIA indicated a lower proportion of plant matter in rudd diet in September than was suggested by GCA (Table 2 and Fig. 1). The SIAR stable isotope mixing model showed 51% and 62% plant matter consumed by rudd in Milada and Most Lakes, respectively. In contrast, SIA showed a higher percentage of animal prey consumption (Milada: 49%, Most: 38%) than the GCA (7.5%, 7%) (Table 2 and Fig. 1). No clear trend was observed between fish size and δ^15^N or fish size and the proportion of plant matter in gut contents (Supplementary Figs S1 and S2). Additionally, there were no statistically significant differences in δ^13^C and δ^15^N between sampling years 2013 and 2014 (see Supplementary Table S1, and Fig. S3 for SIA biplots).

### Results of feeding experiments

**Figure 2:**
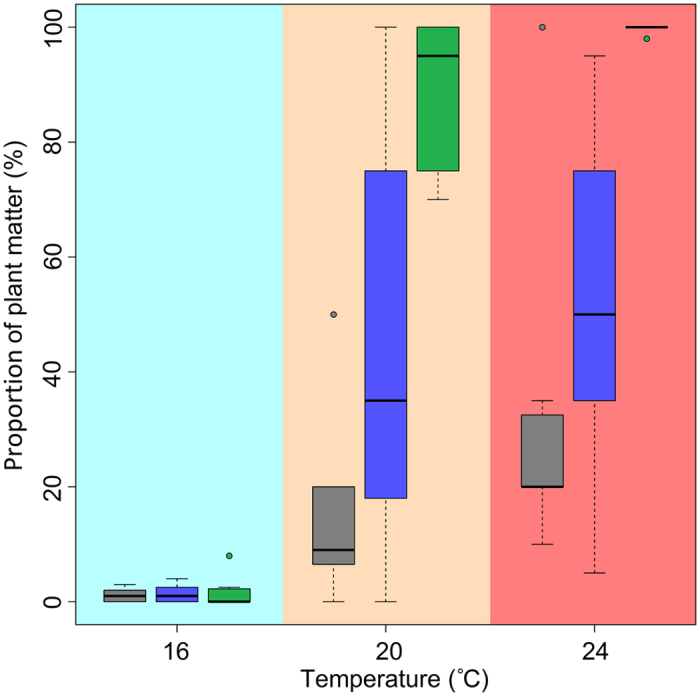
Proportion of plant matter in gut content of experimental rudd in given temperatures and three different diet ratios. The diet ratios of animal prey vs. plant matter were 1:1 (grey), 1:10 (blue) and 1:400 (green). Box and whiskers plots: upper and lower quartiles (boxes), median values (line inside the boxes), maximum and minimum values (whiskers), and outliers (circles) are shown.

Under experimental conditions reflecting the natural conditions in Milada Lake (an animal prey to plant matter ratio of 1:400), plant matter dominated rudd diets at 24 °C (99.8%) and 20 °C (88.8%). In contrast, plant matter constituted an average of only 1.6% of gut contents at 16 °C (Fig. 2 and Table 3). The percentage of plant matter at 24 °C and 20 °C decreased with changing diet ratios. In the presence of a ratio of animal prey to plant matter of 1:10, plant matter comprised 52.5% and 44.5% of the gut contents, respectively. At 16 °C and a ratio of 1:10, the proportion of plant matter remained very low (1.4%). The final experiment used a ratio of animal prey to plant matter of 1:1, which differed the most from natural conditions of all the treatments. Under these conditions, the percentages of plant matter at 24 °C and 20 °C decreased to 31.9% and 15.1%, respectively. At 16 °C and a 1:1 ratio, the proportion of plant matter was only 1.1% (Table 3 and Fig. 2).

Both temperature and the ratio of available food sources significantly affected the diet preferences of rudd. The impact of temperature was greater (F_2,15_ = 10.35, *p* < 0.01) than the impact of diet ratio (F_2,15_ = 2.67, *p* < 0.10). The proportion of plant matter in rudd diet decreased with decreasing temperature. The interaction of temperature and diet ratio also had a statistically significant impact (F_8,8_ = 31.11, *p* < 0.001).

At 16 °C, all studied rudd exhibited very similar feeding behaviour regardless of diet ratio, and the percentage of plant matter consumed was consistently very low (an amount greater than 4% was found in only one individual). At 20 °C, food consumption was more variable, and we found individuals with digestive tract contents ranging from 100% plant matter to 100% animal prey. At 24 °C, the variability was lower than at 20 °C, with the highest variability found when using a 1:10 diet ratio (Table 3 and Fig. 2).

In the additional experiment with only animal prey available as a food source at 24 °C, all eight individuals had digestive tracts full of animal prey (100%). In contrast, in the trial with only plant matter available as a food source and a temperature of 16 °C, the digestive tracts of all ten individuals were entirely empty (even when using the spare individuals, see Methods). In the final additional experiment with only plant matter as a food source at 13 °C for 168 h (*i.e.,* assuming major starvation), all eight individuals utilized plant matter (100%; Table 3) and the specimens’ entire digestive tracts were evenly filled with the plant material.

A slight difference in mass was observed before and after experiments for all rudd individuals. In most cases (except for the two trials discussed below), the slight mass increases were caused by the filling of the digestive tract. There was virtually no difference observed after subtracting the mass of gut contents. In the experiments with both animal prey and plant matter, the mean difference ± SD was 0.35 g ± 0.24 and was statistically insignificant for all experiments (*p* > 0.1). In the case of experiments with only plant matter available at 16 °C, a slight decrease in mass was observed (mean ± SD: 0.06 g ± 0.05) but it was also statistically insignificant (F_1,10_ = 1.13, *p* > 0.1). A greater decrease in mass was observed in the experiment with only plant matter at 13 °C. In this trial, the mean decrease was 0.75 g ± 0.32, which corresponds to a mean mass decrease of 2.7% of total mass and was statistically significant (F_1,8_ = 5.4, *p* = 0.02).

## Discussion

Based on the gut contents of rudd in both lakes in September (surface water temperature: 19.1–21 °C), rudd show a tendency towards herbivory during summer when a sufficient amount of macrophyte plant material is available. The results for Milada Lake demonstrate that this is also true for juvenile rudd, which contradicts the work by Nurminen *et al*.^34^, who observed that rudd did not utilize a plant diet during their first year. However, if the only plant matter available is periphyton and/or detritus juvenile rudd ignore it and utilize zooplankton, as shown by the results for Most Lake. In contrast, older rudd consume periphyton and detritus extensively in the absence of macrophytes. When a sufficient number of macrophytes are present, periphyton and detritus are ignored; this was demonstrated by the results for Milada Lake.

Gut content analyses conducted in May (surface water temperature: 13.1–14.2 °C) show that the rudd completely disregarded plant matter even though macrophytes and periphyton were present. The observed tendency towards switching between food sources is consistent with other studies on rudd. Based on our results, temperatures approximately 20 °C seem to be a crucial threshold for transitioning to herbivory^26^,^30^,^31^. This trend is not only valid in our chosen model organism but can also be observed in other species. For example, a study focused on a rudderfish (*Girella nigricans*) showed that they exhibited the highest RNA:DNA ratio (the most intensive growth in a short-term period) when they utilized plant matter at temperatures above 22 °C. Whereas at 17 °C, a low RNA:DNA ratio indicated that fish were experiencing stress while utilizing plant matter^14^. According to the SIA conducted for rudd in September, the percentage of plant matter consumption was lower than that shown by GCA. This difference is likely caused by isotope turnover in fish tissue^35^. The tissue of rudd still contained an isotopic signal from food assimilated during colder periods, when animal prey was preferred. Nevertheless, we cannot exclude the possibility that the isotopic signal was enriched by the assimilation of bacteria responsible for cellulose digestion, rather than by the plants themselves^36^.

The experimental portion of our study provided support for the hypotheses that both temperature and availability of food play important roles in the food preferences of rudd. Rudd were almost entirely herbivorous in conditions similar to those of Milada Lake, represented by trials with the highest ratio of plant matter to animal prey and temperatures of 20 °C and 24 °C. Decreasing plant matter and increasing animal prey led to a decrease in observed herbivory. This trend was more apparent at 20 °C than at 24 °C. Thus, plant matter seems to be more readily utilized in warm water when it is readily available.

The critical point for transition to plant utilization was observed at 16 °C. Rudd essentially consumed only animal prey at this temperature in all three experimental concentrations of animal prey and plant matter. Interestingly, in the experiment at 16 °C which provided only plant matter, the rudd preferred to consume no food rather than to consume only plant matter. It should be emphasized that this occurred after a period of starvation lasting four days. Such a strategy has been observed among carnivorous fish but is not common for omnivorous or herbivorous species^22^.

These findings indicate that low temperature is the key factor driving the elimination of plant matter from the diet of rudd. This effect does not only apply to fish but is also observed in invertebrate omnivores such as copepods^37^ and snails (*Lymnaea stagnalis*)^38^. Preference for plant matter decreases rapidly when the temperature falls below 20 °C. Plant matter is completely ignored when the temperature falls to 16 °C, which likely explains why Dorenbosch and Bakker^9^ noted low preference for plant matter among rudd and grass carp (*Ctenopharyngodon idella*). The strong preference for animal prey observed by Dorenbosch and Bakker^15^ was likely influenced by the low temperature in their experiment (18 °C), and by the ratio of available animal prey to plant matter (2.9:1). These results are consistent with our findings that rudd in both lakes utilized only animal prey in the spring, when the surface water temperature ranged from 13.1 to 14.2 °C. Piscivory was even observed in rudd during the spring, in low temperatures in the Niagara River^26^.

In extreme cases, fish are likely capable of gaining a minimal amount of energy from plant sources even in cold water, as shown in when rudd started to utilize plant matter at 13 °C after 168 h of starvation in one of our experiments. This suggests that fish are able to ingest plant matter at low temperatures, but this behaviour is not sustainable in the long-term. The mean decrease in mass of rudd individuals in our experiment was 2.7%, supporting the theory of Behrens and Lafferty^14^ that fish begin to experience stress at temperatures below 17 °C when consuming plant matter. However, exceptions were discovered in Antarctica, where extreme herbivory is found in four species of notothenioid fishes (Notothenioidei), which utilize algae^19^, and references therein.

To better understand the observed trends related to temperature, it is necessary to understand how fish digest plant matter. Fish lack specific organs for plant matter digestion, such as the specialized stomachs found in herbivorous mammals and some birds^10^,^39^. Therefore, to use cellulose as a nutrient source they require the enzyme cellulase. B-1,4 glycosidic bonds must be cleaved to release glucose units^40^. For this process, fish depend on the production of enzymes by symbiotic microorganisms living in their digestive tracts^10^,^11^. The microorganisms enter the digestive tract via consumed food, primarily through detritus^10^,^31^,^41^. The cellulase levels in the digestive tracts of rudd are typically highest in late summer and lowest in early spring^10^. This is known from the work by Saha *et al*.^42^, who isolated a series of *Bacillus circulans* and *B. megaterium* from grass carp and tilapia (*Oreochromis mossambica*). A characterization of the isolated microorganisms revealed that they could grow in a wide range of pH levels (5–11) and temperatures (15–42 °C). Similarly, a wide temperature range was found to be suitable for microorganisms with cellulase isolated from the cyprinid *Labeo rohita*^43^. Therefore, the cellulase produced by microorganisms capable of living in the digestive tracts of ectotherms can function at lower temperatures in comparison to other water organisms, where optimum temperatures for cleaving complex carbohydrates range from 30–60 °C^44^,^45^,^46^. Although the lower limit for cellulose functioning in microorganisms is 15 °C, herbivory by fish still seems to be unbeneficial from 16–19 °C, (based on our observation^26^,^30^,^32^), probably due to low cellulase activity. Therefore, the precise temperature at which plant diet starts to be ignored by fish will vary by species and individual.

However, a temperature of approximately 15 °C is still relatively high for an aquatic ecosystem. At our study site (Milada Lake: 50.4°N), the surface water temperature is warmer than 15 °C for only 149 days (41%) each year on average (Palivový kombinát Ústí s.p., unpubl. data). The period of effective cellulose digestion is dramatically shortened with increasing latitude. This is likely the reason that species richness in omnivorous fish decreases dramatically above 55°, and why they are absent above 60°^16^. This trend is stronger in freshwater than in marine ecosystems, probably due to ocean currents that influence water temperature^16^. Warm currents can increase the temperature at higher latitudes, and the opposite can occur at lower latitudes. In contrast to marine conditions, in freshwater the temperature is more strictly correlated with latitude. The trend of decreases in the number of species utilizing plant matter at higher latitudes is also stronger for fish than for terrestrial ectotherms, such as reptiles^16^,^47^. This is likely due to differences in their habitats. In cold climates, reptiles have solved the problem of plant digestion by reducing their size. A small body can be more quickly warmed by air, especially with a warmed under layer that enables cellulase activity. This strategy is not viable in water ecosystems, which have a high specific thermal capacity.

Although these trends in fish herbivory are slightly less apparent in marine than in freshwater ecosystems, the poleward movement of herbivorous species in marine ecosystems due to climate change has already had an apparent negative impact. For example, losses of kelp forests caused by increases in herbivorous consumers have been observed^24^,^25^,^48^,^49^. The spread of herbivorous species in freshwater ecosystems has likely not been extensively studied to date. Nevertheless, we can assume that these changes are occurring. The impact of non-native fish species on macrophytes is evident as observed in studies regarding the introduction of rudd in New Zealand and North America^27^,^28^. We can also presume that fish popular for use in aquaculture, such as *Tilapia* spp. or grass carp^9^,^50^, are likely to spread by human activity and will have negative impacts on macrophytes.

In conclusion, temperature is the cause of fish developing strict herbivorous specializations only in tropical regions, except for a few extreme cases as mentioned above. At higher latitudes, omnivorous fish only utilize plant matter^13^,^14^, which is restricted by temperatures lower than 15 °C. Herbivorous fish need a regular intake of food; therefore an annual decrease in water temperature below 15 °C, even for a short time period, prevents fish speciation and herbivorous specialization at higher latitudes. Climate change is expected to have stronger impacts on water temperatures at higher latitudes, allowing the dispersion of herbivorous feeding behaviours by fish. This can benefit many omnivorous fish species and greatly shape the future of fish communities in lakes of higher latitude regions.

## Methods

### Study site

**Figure 3:**
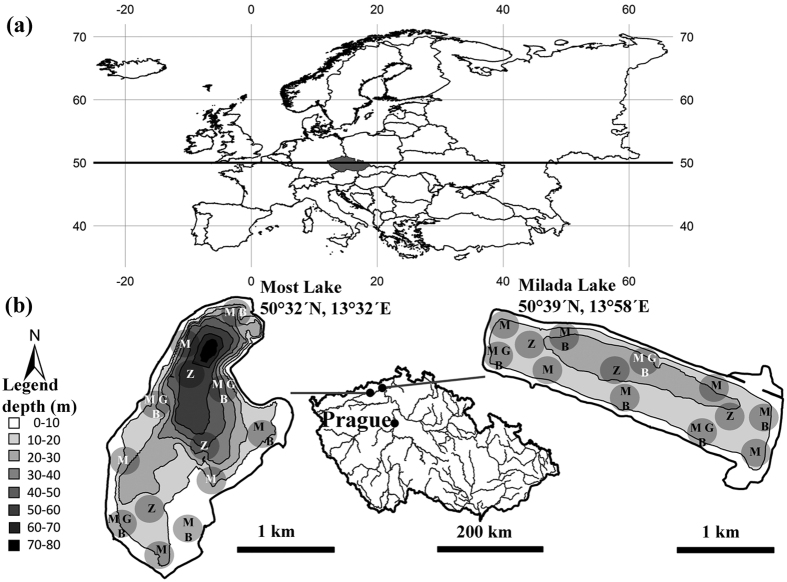
A map showing (**a**) the location of the Czech Republic within Europe (latitude of 50° north as bold line) and (**b**) the location of the two study sites, Milada and Most Lakes, in the Czech Republic. A detailed view of the bathymetric maps shows contour lines with relevant depths and sampling localities: B = benthic invertebrates sampling; G = gillnets sampling; M = macrophytes sampling; Z = zooplankton sampling. The figure was generated by the software ArcMap, version 10.2.2^59^.

The study was conducted in two newly created opencast mine lakes, Milada Lake (50°39′N, 13°58′E) and Most Lake (50°32′N, 13°32′E), in the Czech Republic (Fig. 3). The oligotrophic to mesotrophic Milada Lake has a mean summer total phosphorus (TP) in the surface layer of <10 μg L^−1^, and is located 80 km northwest of Prague (Fig. 3b). It has an area of 250 ha, a volume of 0.036 km^3^ and a maximum depth of 25 m. Aquatic restoration started in 2001 and was finished in 2011. Several species of macrophytes and algae are present at high levels of biomass to a depth of 12 m^51^. The biomass of rudd was 3.58 kg ha^−1^ in 2013, and 2.50 kg ha^−1^ in 2014. Other fishes present are perch (*Perca fluviatilis*), roach, ruffe (*Gymnocephalus cernua*), pike (*Esox lucius*), European catfish (*Silurus glanis*), tench (*Tinca tinca*) and pikeperch (*Sander lucioperca*). The oligotrophic Most Lake (TP < 5 μg L^−1^) is 75 km northwest of Prague. It has an area of 310 ha, a volume of 0.07 km^3^ and a maximum depth of 75 m. Aquatic restoration started in 2008 and was finished in 2014. Macrophytes were not abundant in 2014. The biomass of rudd was 1.9 kg ha^−1^ in 2013 and 0.6 kg ha^−1^ in 2014. Other fishes present are perch, roach, ruffe, pike, European catfish, tench and maraena whitefish (*Coregonus maraena*).

Water temperature and dissolved oxygen were measured using a calibrated YSI 556 MPS probe (YSI Incorporated - Yellow Springs, Ohio, USA) in both lakes, three times each during sampling in September 2014 and May 2015.

### Collection of plant and invertebrate samples

To obtain a qualitative assessment of macrophytes, two SCUBA divers visually assessed their occurrence at ten transects. Transects were marked from the shore to a depth of 12 m in both lakes in September 2013, September 2014 and May 2015 (Fig. 3b), using measuring tapes. The coverage of each macrophyte species, the uncovered bottom area, the percentage composition of each species, and the percentage of uncovered bottom area were measured at 1 m depth intervals. The results of duplicate measurements were averaged for a more accurate assessment. To obtain a quantitative assessment of macrophytes, SCUBA divers staked a quadrangle (50 × 50 cm) at six locations at a depth of 0–3 m (Fig. 3b) with 100% macrophyte coverage. All macrophytes and macroalgae were removed from the area, put into a sack, raised to the boat and weighed after being allowed to drain for 10 minutes. *Potamogeton* spp., *Chara* sp., *Vaucheria* and *Elodea canadensis* were determined to be potentially palatable to rudd, according to past studies^9^,^15^,^30^ and our observations. *Myriophyllum* sp. was classified as an unpalatable species and was not included as a potential food source for rudd. The mean coverage of palatable species per 1 m^2^ at a depth of 0–3 m (where most rudd were found by SCUBA divers and in gillnet catches) was estimated using both quantitative and qualitative information. Samples of palatable macrophytes were collected and frozen for stable isotope analysis. To obtain information about periphyton, the stony shoreline was assessed visually from the boat using polarized glasses.

Sampling was conducted for benthic invertebrates (animal prey), at a depth of 0–3 m in both lakes in September 2014. SCUBA divers sampled six locations evenly distributed along the lake; these were identical to the locations for quantitative assessment of macrophytes (Fig. 3b). At each location a plastic corer (length 50 cm, diameter 8 cm) was used to collect two samples of invertebrates living in the fine sediment in three depth zones (0.5, 1.5 and 3 m). All six subsamples were then combined to give one composite sample per locality. For invertebrates living on the stones, a quadrangle (50×50 cm) was staked out, and all macroscopic invertebrates (most of which were waterlouse or zebra mussel specimens) were collected using forceps. Next, all upper stones were raised from the staked area to the boat to collect any remaining individuals. Two subsamples were obtained and combined to give one sample per location. The bottom cover of fine sediment and stones was measured at each location to determine the percent cover of each type of bottom material. In Milada Lake, invertebrates living on macrophytes were also collected from the 50×50 cm staked area. All macrophytes were collected, put into a sack, raised to the boat and rinsed, and all invertebrates were collected using forceps. Two subsamples were obtained and combined to give one sample per location. The previously mentioned two (benthic invertebrates from fine sediment and stones; Most Lake) or three (benthic invertebrates from fine sediment, stones and macrophytes; Milada Lake) samples were combined from each location. All invertebrates were counted, weighed and preserved in 4% formaldehyde for subsequent identification to the species level in the laboratory. To estimate mean biomass of invertebrates per 1 m^2^ of the lake bottom, the mean biomass of invertebrates living in fine sediment and on stones and macrophytes was recalculated proportionally. Subsamples of benthic invertebrates occurring in the environment were collected and frozen for stable isotope analysis.

In both lakes, zooplankton was sampled in triplicate with a plankton net (diameter: 24 cm, mesh size: 100 μm) at three locations by a vertical haul at 0–20 m in September 2014 (Fig. 3). The samples were fixed with 4% formaldehyde. In the laboratory, the organisms were identified to order or genus and counted under the microscope (Olympus CX40, 100 magnifications) using a Sedgewick-Rafter chamber. *Daphnia*, the taxa most vulnerable to fish predation, were divided into the following two groups: small (<700 μm carapace length) and large (≥700 μm). The reported density of zooplankton is the mean of all nine samples. Zooplankton samples were collected and frozen for stable isotope analysis in March, May, July, September and November 2014.

### Fish sampling

All animal handling (including fish sampling, GCA, SIA and the mesocosm experiment) was performed according to the guidelines of and with permission from the Experimental Animal Welfare Commission at the Ministry of Agriculture of the Czech Republic (Ref. No. CZ 01679). The Experimental Animal Welfare Commission approved all experimental protocols.

In both lakes, fish were sampled for GCA and SIA using benthic multi-mesh gillnets following the European standard^52^. Gillnets were set overnight; they were placed in the water 2 h before sunset and lifted 2 h after sunrise. Sampling was conducted three times each at depths of 0–3, 3–6 and 6–9 m at three locations. Altogether, 72 gillnets were set in both lakes and both in 2013 and 2014. All captured rudd were anaesthetised using a lethal dose of tricaine methanesulfonate (MS–222, Sigma Aldrich Co.). Rudd for the experiment were also sampled by electrofishing (600 W-pulsed DC current) in Milada Lake in September 2015.

In September 2013 and 2014, 80 rudd older than one year and 30 juveniles were used for GCA. In May 2015, 25 individuals older than one year were used; in all cases, the samples were from both lakes. The analysis was conducted on the same day as sampling. The mean sizes of the adult rudd used for GCA were 281.7 mm ± 92.4 (SD) and 191.3 mm ± 38.9 for Milada and Most Lakes, respectively. The mean mass measurements were 100.8 g ±.56.5 and 515.9 g ±.477.4, respectively. The mean sizes of the juvenile rudd were 65 mm ±2.6 and 63.8 mm ± 2.4, and the mean mass measurements were 1.8 g ± 0.4 and 1.2 g ± 0.5 for Milada and Most Lakes, respectively. Fish scales were collected and used to age the fish. After collection, 76 rudd from Milada Lake and 56 rudd from Most Lake in September 2013 and 2014 were frozen to preserve them for SIA. The mean sizes of the rudd used for SIA were 271.4 mm ± 98.6 and 184.8 mm ± 47.2 for Milada and Most, respectively, and the mean mass measurements were 476.3 g ± 467.9 and 94.6 g ± 66 for Milada and Most, respectively.

### GCA

For rudd obtained from the experiment and the lakes, gut contents were identified under a dissecting microscope and the percent composition of their diet by volume was visually estimated. The diet of experimental rudd was categorized into two groups based on what they were offered during the experiment (plant matter or animal prey). The diet of rudd obtained from the lakes was categorized into seven functional groups (macrophytes, periphyton, detritus, benthos, zooplankton, *Dreissena* and the aerial stage of aquatic insects). For the experimental individuals, the gut contents (wet mass) were also weighed (Kern CKE 3600–2, accuracy = 0.01 g) to determine wet mass.

### SIA of fish and potential food sources

Dorsal muscle tissue was dissected from individual fish from both lakes and cleaned of scales and skin. All samples (fish and diet samples) were washed with distilled water. The samples were dried at 60 °C for 48 h and ground into a homogenous powder using a ball-mill Retsch MM 200 (Retsch GmbH, Haan, Germany). Small subsamples (0.520–0.770 mg) were weighed in tin cups for the analysis of δ^13^C and δ^15^N. The isotope analyses were performed using a Thermo Finnigan DELTA^plus^ Advantage continuous flow stable isotope-ratio mass spectrometer, connected to a Carlo Erba Flash EA1112 elemental analyser (Thermo Electron Corporation, Waltham, MA, USA) at the University of Jyväskylä, Finland. Measurements of δ^13^C and δ^15^N were reported in per mil (‰) notation, using internal standards with a known relationship to the international standards of Vienna Pee Dee belemnite (for carbon) and atmospheric N_2_ (for nitrogen). Sample analyses also yielded the elemental composition of carbon and nitrogen (by mass). Because the C:N ratios were consistently lower than 3.5, isotope values were not lipid corrected^53^.

### Feeding experiments

From September 14 to 24, 2015, 12 different experiments were conducted and duplicated on the shore of Milada Lake, using different diet ratios and water temperatures. Each experiment lasted 24 h, except for one that lasted 168 h (Table 4). Fibreglass tanks were placed under a tent pavilion to prevent contact with rain and sunlight, and were filled with water from Milada Lake filtered through a sieve (mesh size 170 μm) (tank diameter = 1 m, surface of tank bottom = 0.785 m^2^, total height of the tank = 0.8 m, height of water column = 0.64 m and total water volume in the tank = 502 L). During the experiments, water temperatures were measured at 4 h intervals, and the temperature was maintained with a maximum deviation of ± 0.6 °C. Oxygen volume was also measured at the same time intervals (ranging from 11.7 to 6.8 mg L^−1^ with the highest and lowest values at 13 °C and 24 °C, respectively). Water was heated or cooled to the required temperature using aquarium heaters (Eheim Jäger 300 W) and freezer packs containing refrigerant gel.

Diet ratios for the experiments were selected according to the occurrence of palatable plants and animal prey in the lake (see above and Table 1) estimated to the tank surface area. The estimated available ratio (by wet mass) of animal prey to plant matter in Milada Lake was 1:395.2, presented as 1:400 for simplification. Then, diet masses corresponding with each of the required ratios (1:10, 1:1, 1:0, 0:1) were calculated for each experiment (Table 4). *Potamogeton pectinatus* from Milada Lake and *Chironomus* sp. from cultivation were placed in the required amounts on the tank bottom. Both types of diet have been shown to be readily utilized by rudd. Prior to placement in the tank, macrophytes were rinsed properly to remove invertebrates, and the required mass was weighed after being allowed to drain for 10 minutes. Live *Chironomus* sp. were placed in shallow plastic buckets (25 × 25x 10 cm) to prevent them from hiding.

Three days prior to the experiment, the rudd were caught from Milada Lake by electrofishing. The rudd were starved for one day in cage nets in the lake, and further acclimatized for two more days at the selected temperatures in extra tanks without food. The rudd individuals were measured, weighed and placed into the experimental tanks 4–6 h after the appropriate diet was added to the tanks. Five rudd individuals were used for each experiment (120 fish in total). After the experiment, the rudd were anaesthetised using a lethal dose of MS–222, weighed, and dissected for gut contents, which were also weighed to acquire a measurement of wet mass. Four individuals were analysed and one was kept as a spare for cases where individuals were empty. The mean size of rudd used for gut content analysis in the feeding experiments was 137.3 mm ± 17.6 (SD), and the mean mass was 29.4 g ± 9.3 (SD).

### Statistical analysis

The effects of temperature and diet ratio on rudd dietary preferences in our experiments were tested using a general linear model and a split-plot design. The observed percentage of plant diet of each fish was tested as nested in the tank variable, which was set as a random factor. The arcsine square root transformation was used to improve non-normal distribution of percentage data. One-way ANOVA was used to test for differences in fish mass before and after the experiment. Dependency between fish size and δ15 N, and between fish size and proportion of plant matter in gut content was tested by a linear regression analysis. A nonparametric Kruskal–Wallis test (Statistica 12; Stat-Soft Inc., Tulsa, OK) was used to test for differences in δ^13^C and δ^15^N between years 2013 and 2014. The freeware package SIAR 4.0 for linear mixing models was used to determine the ratio of potential food sources to assimilated diet in both lakes^54^,^55^. Means ± SD of δ^13^C and δ^15^N from samples were entered into the model. Concentration dependence was included in the form of proportions of C and N in food sources^56^ because these concentrations differ markedly between plant and animal samples (*cf*. Dataset). For carbon, we used a trophic fractionation factor of 0.4^57^. For nitrogen, we used 3.4 and 4.8 for animal prey and plant matter, respectively^57^,^58^.

## Additional Information

**How to cite this article**: Vejříková, I. *et al*. Distribution of Herbivorous Fish Is Frozen by Low Temperature. *Sci. Rep.* **6**, 39600; doi: 10.1038/srep39600 (2016).

**Publisher's note:** Springer Nature remains neutral with regard to jurisdictional claims in published maps and institutional affiliations.

## Supplementary Material

Supplementary Information

Supplementary Dataset

## Figures and Tables

**Table 1:** Summary of the assessment of potential diet for rudd in Milada and Most Lakes.

	Depth 0–3 m						Depth 0–20 m		
	Macrophyte cover.(%)		Depth 0–3 m Mean biomass of potential sources (g m^−2^)				Depth 0–20 m Mean density (ind. L^−1^)		
	Palatable macroph.	Unpalatable macroph.	Palatable macroph.	Unpalatable macroph.	Benthic invert.	*Dreissena*	Copepods	Cladocerans (*Daphnia*)	
								small	large
Milada	71	20	2122	598	5.4	167	23.5	10.1	2.7
Most	0.9	0.8	27	24	4.2	242	0.3	26.8	0.3

We assessed mean coverage and mean biomass of palatable and unpalatable macrophytes on the bottom in the depth of 0–3 m, and biomass of benthic invertebrates in the same depth. Further, we assessed mean density of zooplankton in the water column in the depth of 0–20 m.

**Table 2:** Mean proportion of diet categories (% ± standard deviation) in Milada and Most Lakes during September and May according to gut content analysis.

Date	Lake	N	Proportion of given diet category in gut content, mean ± SD (%)						
			Macroph.	Periphyton	Detritus	Benthos	Zoopl.	*Dreissena*	A. insect
September	Milada	80	92.5 ± 22	0	0	7.5 ± 22	0	0	0
2013 and 14	Most	80	0	68 ± 34	25 ± 31	7 ± 13	0	0	0
May 2015	Milada	25	0	0	0	0	0	0	100
	Most	25	0	0	0	42 ± 41	13 ± 20	0	45 ± 40

The size range of rudd was 98–430 mm (TL; >1 year old). N = number of dissected individuals, Zoopl. = zooplankton and A. insect = aerial stage of aquatic insect.

**Table 3:** Mean proportion of plant matter in gut content of experimental rudd (% ± standard deviation) for each experiment in given diet ratios (animal prey vs. plant matter) and temperatures.

T, Time	Repetition no.	Proportion of plant matter in given diet ratio (%)				
		1:400	1:10	1:1	0:1	1:0
13 °C, 168 h	1	—	—	—	100 ± 0	—
13 °C, 168 h	2	—	—	—	100 ± 0	—
16 °C, 24 h	1	2 ± 4	1.3 ± 1.5	1.3 ± 1.5	Empty	—
16 °C, 24 h	2	1.1 ± 1.3	1.5 ± 1.9	1 ± 1.2	Empty	—
20 °C, 24 h	1	87.5 ± 2	42.5 ± 41.9	14 ± 6.9	—	—
20 °C, 24 h	2	90 ± 14.1	46.5 ± 36.5	16.3 ± 22.8	—	—
24 °C, 24 h	1	100 ± 0	36.3 ± 21.4	23.8 ± 7.5	—	0 ± 0
24 °C, 24 h	2	99.5 ± 1	68.8 ± 27.8	40 ± 40.8	—	0 ± 0

“Empty” denotes situations when fish were found with no gut contents, whereas 0% of plant matter indicates presence of animal prey.

**Table 4:** Summary of conducted experiments, the diet ratios used (animal prey vs. plant matter) and mass ratios counted, temperatures, duration and dates of the experiments.

Diet ratio	Mass ratio (g)	Duration (h)	Date of the experiment in 2015			
			T = 13 °C	T = 6 °C	T = 20 °C	T = 24 °C
1:400*	4.2:1,666	24	—	Sept. 14	Sept. 14	Sept. 14
1:10	152:1,518	24	—	Sept. 15	Sept. 15	Sept. 15
1:1	835:835	24	—	Sept. 16	Sept. 16	Sept. 16
1:0	1,670:0	24	—	—	—	Sept. 17
0:1	0:1,670	24	—	Sept. 17	—	—
0:1	0:1,670	168	Sept. 18–24	—	—	—

The experiments were conducted always in two repetitions. Note that the total usable diet mass was ca. 1,670 g per tank in all experiments. ^*^ real lake diet ratio, Sept. means September.

## References

[b1] Hutchinson G. E. A Treatise in Limnology. III. Limnological Botany. Wiley Interscience, New York (1975).

[b2] Hutchinson G. E. Thoughts on aquatic insects. Bioscience 31, 495–500 (1981).

[b3] Newman R. M. Herbivory and detritivory on freshwater macrophytes by invertebrates: a review. J. N. Am. Benthol. Soc. 10, 89–114 (1991).

[b4] Lodge D. M. Herbivory on freshwater macrophytes. Aquat. Bot. 41, 195–224 (1991).

[b5] Wood K. A. . Herbivore regulation of plant abundance in aquatic ecosystems. Biol. Rev. online early, doi: 10.1111/brv.12272 (2016). 27062094

[b6] Bakker E. S. . Herbivory on freshwater and marine macrophytes: A review and perspective. Aquat. Bot. online early, doi: 10.1016/j.aquabot.2016.04. 008 (2016).

[b7] Lacoull P. & Freedman B. Environmental influences on aquatic plants in freshwater ecosystems. Environ. Rev. 14, 89–136 (2006).

[b8] Miller S. A. & Crowl T. A. Effects of common carp (*Cyprinus carpio* L.) on macrophytes and invertebrate communities in a shallow lake. Freshwater Biol. 51, 85–94 (2006).

[b9] Dorenbosch M. & Bakker E. S. Effects of contrasting omnivorous fish on submerged macrophyte biomass in temperate lakes: a mesocosm experiment. Freshwater Biol. 57, 1360–1372 (2012).

[b10] Niederholzer R. & Hofer R. The adaptation of digestive enzymes to temperature, season and diet in roach *Rutilus rutilus* L. and rudd *Scardinius erythrophthalmus* L. Cellulase. J. Fish Biol. 15, 411–416 (1979).

[b11] Prejs A. & Blaszczyk M. Relationships between food and cellulase activity in freshwater fish. J. Fish Biol. 11, 447–452 (1977).

[b12] Gerkings S. D. Feeding ecology of fish. Academic Press Inc., San Diego, 416 pp (1994).

[b13] Zimmerman L. C. & Tracy C. R. Interactions between the environment and ectothermy and herbivory in reptiles. Physiol. Zool. 62, 374–409 (1989).

[b14] Behrens M. D. & Lafferty K. D. Temperature and diet effects on omnivorous fish performance: implications for the latitudinal diversity gradient in herbivorous fishes. Can. J. Fish. Aquat. Sci. 64, 867–873 (2007).

[b15] Dorenbosch M. & Bakker E. S. Herbivory in omnivorous fishes: effect of plant secondary metabolites and prey stoichiometry. Freshwater Biol. 56, 1783–1797 (2011).

[b16] González-Bergonzoni I. . Meta-analysis shows a consistent and strong latitudinal pattern in fish omnivory across ecosystems. Ecosystems 15, 492–503 (2012).

[b17] Behrens M. D. & Lafferty K. D. Geographic variation in the diet of Opaleye (*Girella nigricans*) with respect to temperature and habitat. PLoS ONE 7, e45901 (2012). 23029302 10.1371/journal.pone.0045901 PMC3448717

[b18] Floeter S., Behrens M., Ferreira C., Paddack M. & Horn M. Geographical gradients of marine herbivorous fishes: patterns and processes. Mar. Biol. 147, 1435–47 (2005).

[b19] Clements K. D., Raubenheimer D. & Choat J. H. Nutritional ecology of marine herbivorous fishes: ten years on. Funct. Ecol. 23, 79–92 (2009).

[b20] Mead G. W. A history of South Pacific fishes. Scientific explorations of the South Pacific (ed. by W.S. Wooster), pp 236–251. National Academy of Sciences, Washington DC (1970).

[b21] Ferreira C. E. L., Floeter S. R., Gasparini J. L., Ferreira B. P. & Joyeux J. C. Trophic structure patterns of Brazilian reef fishes: a latitudinal comparison. J. Biogeogr. 31, 1093–106 (2004).

[b22] Arrington D. A., Winemiller K. O., Loftus W. F. & Akin S. How often do fishes “run on empty”? Ecology 83, 2145–51 (2002).

[b23] Gaines S. D. & Lubchenco J. A unified approach to marine plantherbivore interactions II. Biogeography. Annu. Rev. Ecol. Evol. Syst. 13, 111–138 (1982).

[b24] Vergés A. . Tropical rabbitfish and the deforestation of a warming temperate sea. J. Ecol. 102, 1518–1527 (2014).

[b25] Vergés A. Beyond the tropics: ocean warming, shifts in species interactions and the tropicalisation of temperate reefs. Species on the Move (oral presentation), 9–12 February 2016, Hobart, Tasmania (2016).

[b26] Guinan M. E. Jr, Kapuscinski K. L. & Teece M. A. Seasonal diet shifts and trophic position of an invasive cyprinid, the rudd *Scardinius erythrophthalmus* (Linnaeus, 1758), in the upper Niagara River. Aquat. Invasions 10, 217–225 (2015).

[b27] Lake M. D., Hicks B. J., Wells R. & Dugdale T. M. Consumption of submerged aquatic macrophytes by rudd (*Scardinius erythrophthalmus* L.) in New Zealand. Hydrobiologia 470, 13–22 (2002).

[b28] Kapuscinski K. L. . Selective herbivory by an invasive cyprinid, the rudd *Scardinius erythrophthalmus*. Freshwater Biol. 59, 2315–2327 (2014).

[b29] Prejs A. & Jackowska H. Lake macrophytes as the food of roach (*Rutilus rutilus* L.) and rudd (*Scardinius erythrophthalmus* L.). I. Species composition and dominance relations in the lake and food. Ekol. Pol. 26, 429–438 (1978).

[b30] Kapuscinski K. L., Farrell J. M. & Wilkinson M. A. Feeding patterns and population structure of an invasive cyprinid, the rudd *Scardinius erythrophthalmus* (Cypriniformes, Cyprinidae), in Buffalo Harbor (Lake Erie) and the upper Niagara River. Hydrobiologia 693, 169–181 (2012).

[b31] Prejs A. Lake macrophytes as the food of roach (*Rutilus rutilus* L.) and rudd (*Scardinius erythrophthalmus* L.) II. Daily intake of macrophyte food in relation to body size of fish. Ekol. Pol. 26, 537–553 (1978).

[b32] Prejs A. Herbivory by temperate freshwater fishes and its consequences. Environ. Biol. Fish. 10, 281–296 (1984).

[b33] González-Bergonzoni I. . Potential drivers of seasonal shifts in fish omnivory in a subtropical stream. Hydrobiologia 768, 183–196 (2016).

[b34] Nurminen L., Horppila J., Lappalainen J. & Malinen T. Implications of rudd (*Scardinius erythrophthalmus*) herbivory on submerged macrophytes in a shallow eutrophic lake. Hydrobiologia 506–509, 511–518 (2003).

[b35] Vander Zanden M. J., Clayton M. K., Moody E. K., Solomon C. T. & Weidel B. C. Stable isotope turnover and half-life in animal tissues: A literature synthesis. PLoS ONE 10, e0116182, doi: 10.1371/journal.pone.0116182 (2015). 25635686 PMC4321325

[b36] Newsome S. D., Fogel M. L., Kelly L. & del Rio C. M. Contributions of direct incorporation from diet and microbial amino acids to protein synthesis in Nile tilapia. Funct. Ecol. 25, 1051–1062 (2011).

[b37] Boersma M. . Temperature driven changes in the diet preference of omnivorous copepods: no more meat when it’s hot? Ecol. Lett. 19, 45–53 (2016). 26567776 10.1111/ele.12541

[b38] Zhang P., Blonk B. A., van den Berg R. F. & Bakker E. S. The effect of temperature on herbivory by the omnivorous ectotherm snail *Lymnaea stagnalis*. Hydrobiologia online early, doi: 10.1007/s10750-016-2891-7 (2016).

[b39] Day R. . Enzymatic digestion in stomachless fishes: how a simple gut accommodates both herbivory and carnivory. J. Comp. Physiol. 181, 603–613 (2011). 21212962 10.1007/s00360-010-0546-y

[b40] Barr B., Hsieh Y.-L. & Ganem B. W. Identification of two functionally different classes of exocellulases. Biochemistry 35, 586–592 (1996). 8555231 10.1021/bi9520388

[b41] Ganguly S. & Prasad A. Microflora in fish digestive tract plays significant role in digestion and metabolism: a review. Rev. Fish Biol. Fisher. 22, 11–16 (2012).

[b42] Saha S., Roy R. N., Sen S. K. & Ray A. K. Characterization of cellulase-producing bacteria from the digestive tract of tilapia, *Oreochromis mossambica* (Peters) and grass carp, *Ctenopharyngodon idella* (Valenciennes). Aquacult. Res. 37, 380–388 (2006).

[b43] Ghosh K., Sen S. K. & Ray A. K. Characterization of Bacilli isolated from the gut of rohu, *Labeo rohita*, Fingerlings and its significance in digestion. J. Appl. Aquacult. 12, 33–42 (2002).

[b44] Suzuki K.-I., Ojima T. & Nishita K. Purification and cDNA cloning of a cellulase from abalone *Haliotis discus hannai*. Eur. J. Biochem. 270, 771–778 (2003). 12581217 10.1046/j.1432-1033.2003.03443.x

[b45] Zin H. W., Park K. H. & Choi T. J. Purification and characterization of a carboxymethyl cellulase from *Artemia salina*. Biochem. Bioph. Res. Co. 443, 194–199 (2014). 10.1016/j.bbrc.2013.11.085 24291747

[b46] Manavalan T., Manavalan A., Thangavelu K. P. & Heese K. Characterization of a novel endoglucanase from *Ganoderma lucidum*. J. Basic. Microb. 55, 761–771 (2015). 10.1002/jobm.201400808 25895101

[b47] Espinoza R. E., Wiens J. J. & Tracy C. R. Recurrent evolution of herbivory in small, cold-climate lizards: breaking the ecophysiological rules of reptilian herbivory. P. Natl. Acad. Sci. USA 101, 16819–16824 (2004). 10.1073/pnas.0401226101 PMC534712 15550549

[b48] Bennett S., Wernberg T., Harvey E. S., Santana-Garcon J. & Saunders B. J. Tropical herbivores provide resilience to a climate-mediated phase shift on temperate reefs. Ecol. Lett. 18, 714–723 (2015). 25994785 10.1111/ele.12450

[b49] Wernberg T. Hot, heatwaves and herbivores– drivers and feedbacks of changing of species distributions and community reconfiguration. Species on the Move (oral presentation), 9–12 February 2016, Hobart, Tasmania (2016).

[b50] Mathavan S., Vivekanandan E. & Pandian T. J. Food utilization in the fish *Tilapia mossambica* fed on plant and animal foods. Helgoland Mar. Res. 28, 66–70 (1976).

[b51] Čech M. . Location and timing of the deposition of egg strands by perch (*Perca fluviatilis* L.): the roles of lake hydrology, spawning substrate and female size. Knowl. Manag. Aquat. Ec. 403, 1–12 (2011).

[b52] CEN, Water Quality – Sampling of fish with multi-mesh gillnets. EN–14757 (2005).

[b53] Post D. M. . Getting to the fat of the matter: models, methods and assumptions for dealing with lipids in stable isotope analyses. Oecologia 152, 179–189 (2007). 17225157 10.1007/s00442-006-0630-x

[b54] Parnell A. C., Inger R., Bearhop S. & Jackson A. L. Source Partitioning Using Stable Isotopes: Coping with Too Much Variation. PLoS ONE 5, e9672. doi: 10.1371/journal.pone.0009672 (2010). 20300637 PMC2837382

[b55] R Development Core Team, R: A language and environment for statistical computing (2015).

[b56] Phillips D. L. & Koch P. L. Incorporating concentration dependence in stable isotope mixing models. Oecologia 130, 114–125 (2002). 10.1007/s004420100786 28547016

[b57] Post D. M. Using stable isotopes to estimate trophic position: models, methods, and assumptions. Ecology 83, 703–718 (2002).

[b58] Mill A. C., Pinnegar J. K. & Polunin N. V. C. Explaining isotope trophic-step fractionation: why herbivorous fish are different. Functional Ecology 21, 1137–1145 (2007).

[b59] Esri, Working with ArcMap. *ArcGIS Help 10.2.2*. (2016). Available at: http://resources.arcgis.com/en/help/main/10.2/#/Mapping_and_visualization_in_ArcGIS_for_Desktop/018q00000004000000/ (Accessed: 14th September 2016).

