# Supplementary material for: Distribution of Herbivorous Fish Is Frozen by Low Temperature
Source: Sci Rep. 2016 Dec 22;6:39600. doi: 10.1038/srep39600 (PMC5177937; doi:10.1038/srep39600)
Supplement: Supplementary Information [file srep39600-s1.pdf]

Supplementary materials for manuscript:

Distribution of Herbivorous Fish Is Frozen by Low Temperature

Vejříková, I., Vejřík, L., Syväranta, J., Kiljunen, M., Čech, M., Blabolil, P., Vašek, M., Sajdlová, Z., Chung, S.H.T., Šmejkal, M., Frouzová, J., Peterka, J.

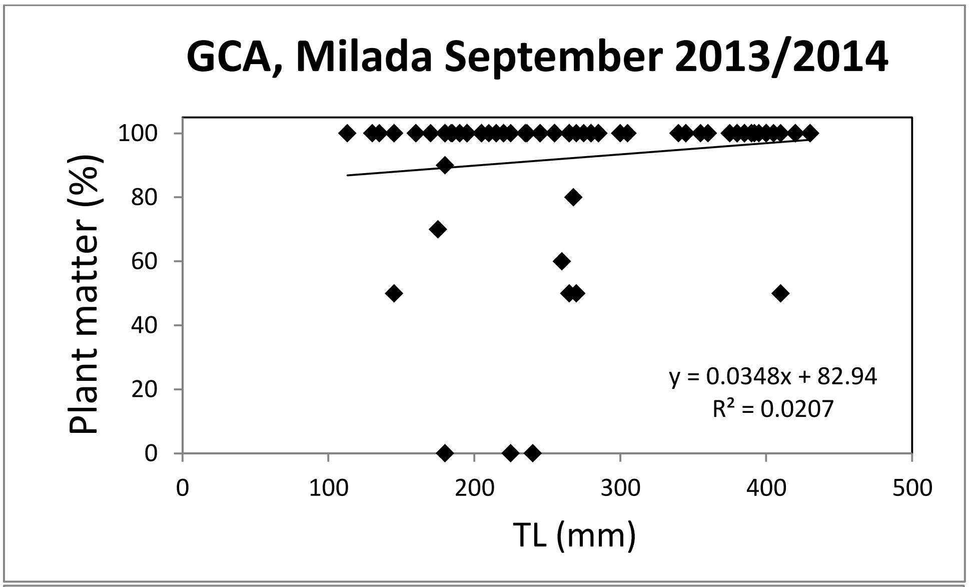

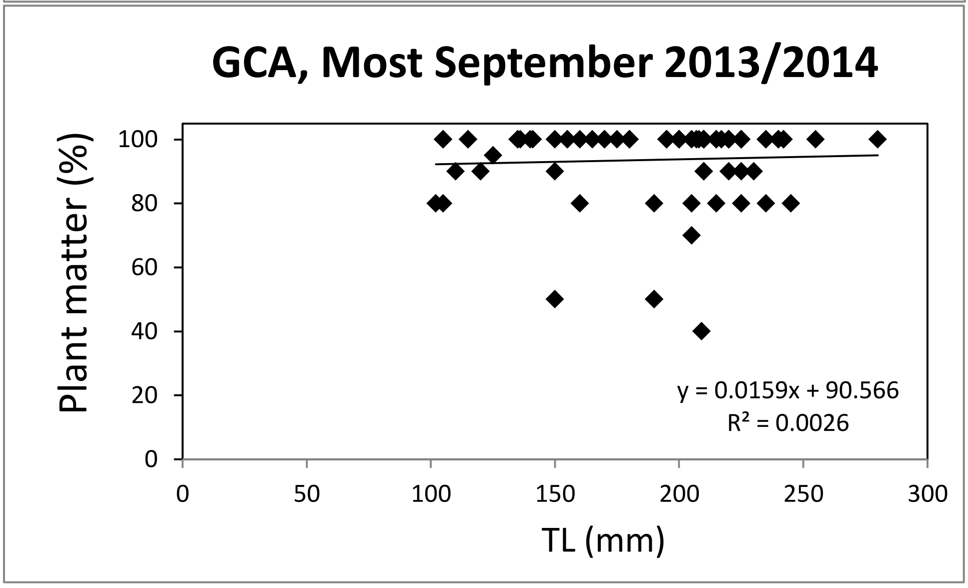

Supplementary Figure S1. Relation between fish size (total length) and proportion of plant matter according to gut content analysis.

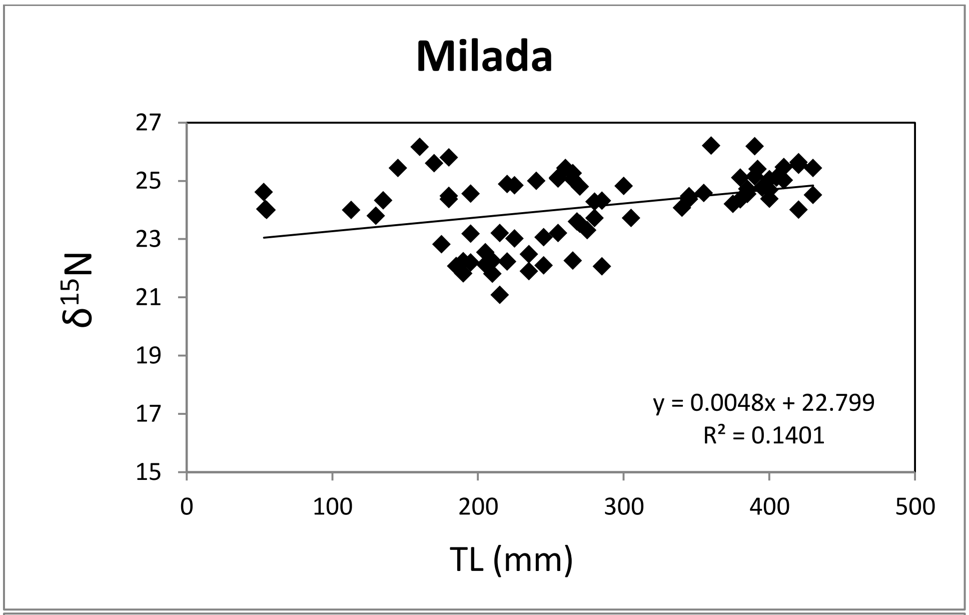

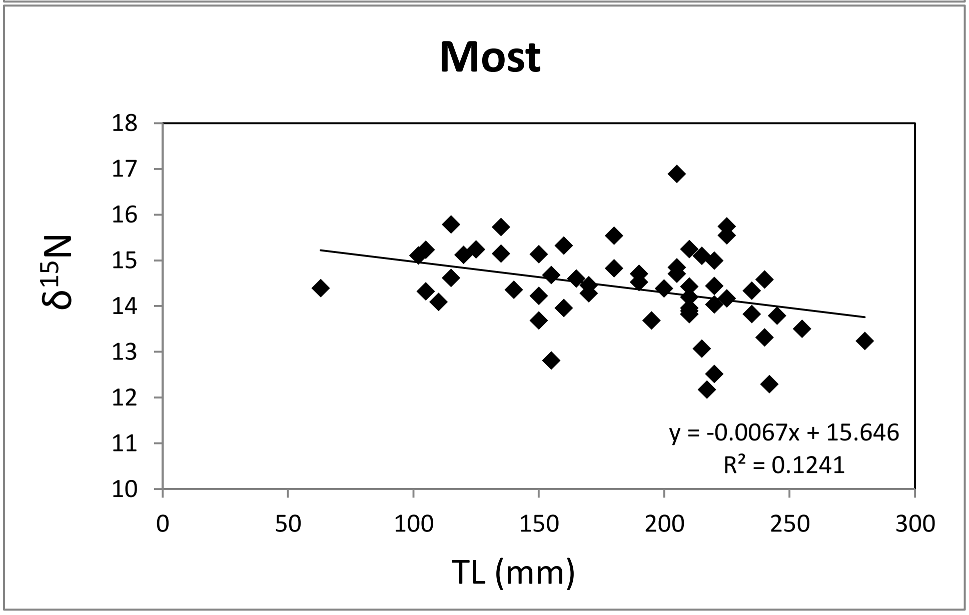

Supplementary Figure S2. Relation between fish size (total length) and $\delta^{15}N$ according to stable isotope analysis.

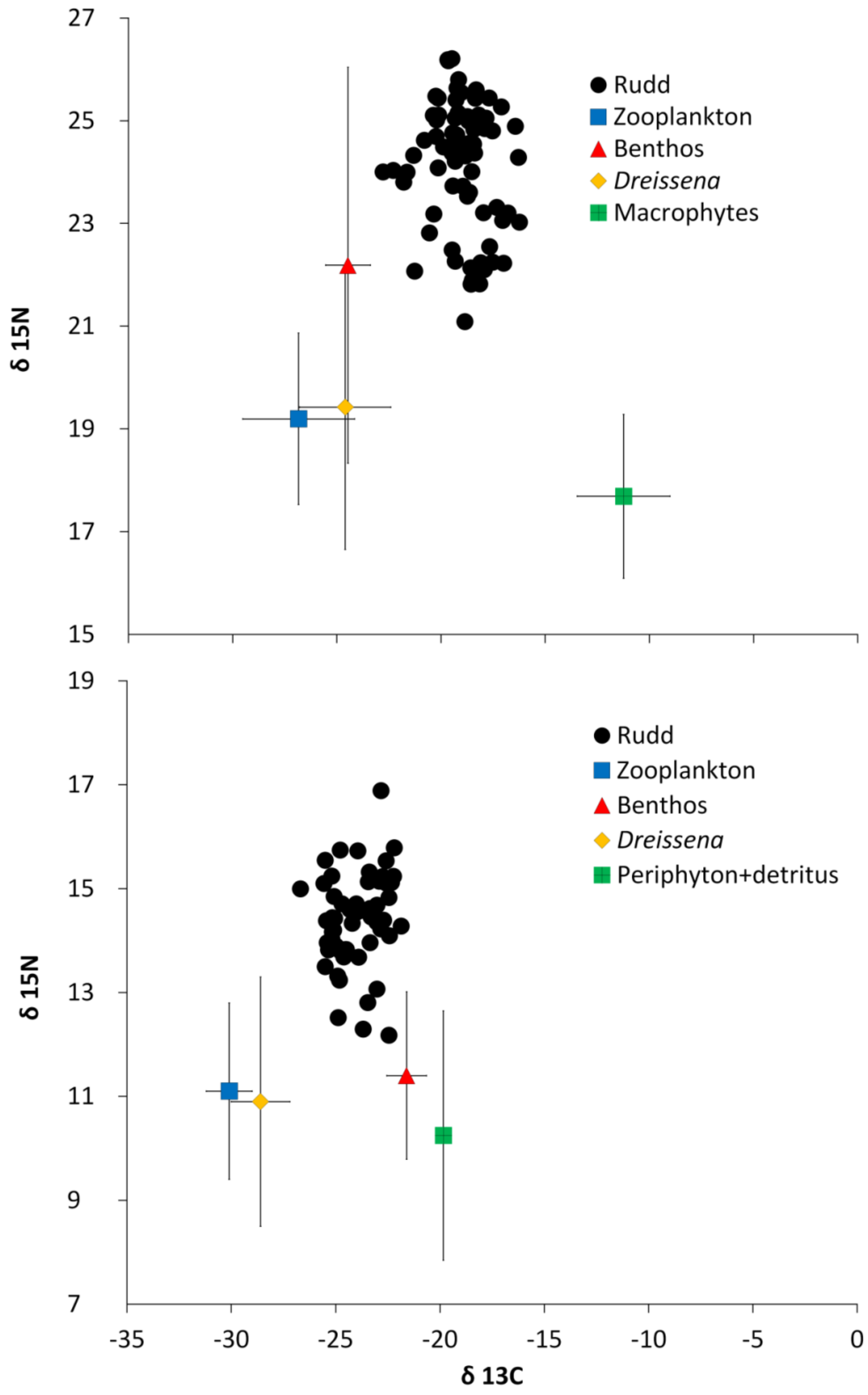

Supplementary Figure S3. Biplots with the data from stable isotope analysis. Upward biplot shows Milada Lake, whereas biplot below shows Most Lake.

Supplementary Table S1. Summary of Kruskal-Wallis test showing no statistical differences in $\delta^{13}C$ and $\delta^{15}N$ between years 2013 and 2014.

	Milada Lake	Most Lake
$\delta^{13}C$	$H_{1,76} = 0.05$ $p > 0.1$	$H_{1,56} = 3.54$ $p > 0.05$
$\delta^{15}N$	$H_{1,76} = 1.32$ $p > 0.1$	$H_{1,56} = 1.81$ $p > 0.1$
